# Trends in application of digital technology in nursing informatics: an integrative bibliometric analysis

**DOI:** 10.3389/fdgth.2026.1670402

**Published:** 2026-02-23

**Authors:** Bomi An, Sujin Choi

**Affiliations:** 1 Department of Nursing, Hannam University, Daejeon, Republic of Korea; 2 College of Nursing, School of Medicine, Soonchunhyang University, Asan, Republic of Korea

**Keywords:** application, digital, informatics, nursing, technolgy

## Abstract

**Background:**

Digital technology has led to innovations in healthcare, particularly in the field of nursing informatics. Although challenges such as resistance to technology, insufficient training, and security have been reported, comprehensive bibliometric analyses evaluating research trends and patterns are scarce. Therefore, this study aimed to examine trends and patterns in the application of digital technologies to nursing informatics by utilizing an integrative bibliometric analysis.

**Methods:**

A comprehensive literature search was conducted on PubMed, CINAHL, MEDLINE, Embase, Web of Science, and Scopus for original articles published before 2024. This review followed the PRISMA guidelines, and 409 studies were included. VOSviewer and Excel 2019 were used to analyze the number of publications by journal, year, country, authors, citations, and keywords.

**Results:**

Digital technology research began in 1985 and has increased significantly since 2015. The United States had the highest number of publications, whereas *Computers, Informatics, and Nursing* had the highest number of publications. Two authors were at the center of the collaboration network. The most frequently used keywords in these studies were virtual reality, nursing, and artificial intelligence. The primary research focus of the top 10 most-cited studies was intervention programs utilizing virtual reality.

**Conclusion:**

This study highlights the growing importance of digital technology in nursing informatics, with research surging since 2015 due to advancements in artificial intelligence, virtual reality, and big data. Issues such as non-standardized nursing practices that utilize digital technologies and ethical considerations remain underexamined. Therefore, nursing professionals should focus on developing digital technology nursing standards in diverse nursing contexts, promote global collaboration, and strengthen digital competencies to maximize the benefits of digital innovation in the field of nursing informatics.

## 1 Introduction

Innovation in digital technology and artificial intelligence (AI) has significantly influenced healthcare by offering opportunities to improve patient care, streamline workflows, and enhance decision-making (1). Digital technologies encompass electronic tools, systems, devices, and resources that produce, store, and process data (2). Applications of digital technologies, such as electronic health records and AI-assisted diagnostic tools, are shaping nursing informatics and improving care efficiency (3). To address these trends, the World Health Organization has released digital health strategies calling for integrated national plans, including workforce and infrastructure targets (4). Based on the digital health strategy, the International Council of Nursing has called for the development of digital technology competencies by nurses, thereby embedding informatics expectations within their professional practice and education (5). In this regard, digital technology has become central to healthcare, particularly in nursing informatics.

Nursing informatics is a core discipline in nursing, computer science, and information management that aims to optimize clinical decision making, education, and research (6). Nursing informatics deals not only with managing healthcare information systems, but also with applying AI technology in practice, education, and health research (7). For instance, nurses documented using an electronic health record system and advanced electronic tools (e.g., telenursing for patients and caregivers’ consultation and education) (8). As such, it has expanded to incorporate advanced technologies and to rapidly reshape healthcare practices by offering patient-specific, efficient, and data-driven solutions (9). However, challenges in digital technology nursing remain prevalent (10–12). Therefore, it is necessary to explore the status of digital technologies in nursing informatics.

The nursing informatics literature has a longstanding tradition of examining various digital technologies in nursing practice, leadership, research, and education, through a framework that evaluates their advantages and disadvantages (13). Key barriers to the implementation and adoption of digital technology nursing include insufficient training (14), increased workload, and low technological confidence, which affect efficiency and quality of care (15). Facilitators include leadership support (14), positive corporate culture, and targeted training initiatives (15). Moreover, the previous literature is concentrated on the types and effectiveness of digital technology nursing, and the challenges and attitudes of nurses toward the use of digital technology in nursing (12).

Although many studies have explored the use of digital technology in nursing, comprehensive bibliometric analyses evaluating the research trends and patterns of applying digital technology in nursing informatics are scarce. Additionally, the existing literature often emphasizes isolated technologies, creating a fragmented understanding and neglecting overarching patterns in digital innovation (14, 16, 17). This study addresses the gap by offering an integrative bibliometric analysis identifying influential studies and underexplored research areas. Bibliometric analysis is useful for interpreting and delineating the accumulated scientific knowledge and developmental trends of established disciplines by rigorously analyzing large volumes of data (18).

By analyzing the scientific literature, this study identifies the trends and patterns in the application of digital technology in nursing informatics, suggesting directions for future research. The specific aims of this study were to (1) identify the number of publications, countries, journals, and authors; (2) discover keywords and the most cited articles. These findings can inform strategies by which nursing professionals can integrate digital technology into their nursing services while moving forward.

## 2 Methods

### 2.1 Study design

This integrative bibliometric review comprehensively examined the application trends and patterns of digital technology in nursing informatics through bibliometric analysis according to the PRISMA guideline process (19).

### 2.2 Data collection (article selection)

Building on Zhu et al.'s recommendations (20) on the technologies utilized in nursing services and education, this study conducted a comprehensive literature search of PubMed, CINAHL, MEDLINE, Embase, Web of Science, and Scopus, which were selected to ensure broad and multidisciplinary coverage of nursing and health-related studies. These databases collectively provide access to the high-quality, peer-reviewed literature and citation data essential for bibliometric analysis.

Data analysis was conducted in January 2024 with no specific time restrictions imposed on the retrieved literature. Studies published in English and indexed in the Social Science Citation Index (SSCI), Scientific Citation Index (SCI), Science Citation Index Expanded (SCIE), SCOPUS, and Arts and Humanities Citation Index (A&HCI) were selected for the analysis, as these citation indexes encompass reputable, peer-reviewed journals and offer consistent citation metrics necessary for bibliometric analysis. Consultations were sought from specialists in the AI field to ensure the appropriateness of the keywords used in the literature search. The primary search terms included “Artificial Intelligence,” “Nursing Informatics,” “Machine Learning,” “Digital,” and “Technology” Supplementary Appendix A.

The collected literature was reviewed using the EndNote software. To minimize potential bias during the review process, two researchers independently screened the studies according to the predefined inclusion and exclusion criteria, and any discrepancies were resolved through discussion. A standard screening protocol was used to ensure consistency and transparency during study selection. Multiple major databases were comprehensively searched to minimize publication selection bias.

**Figure 1:**
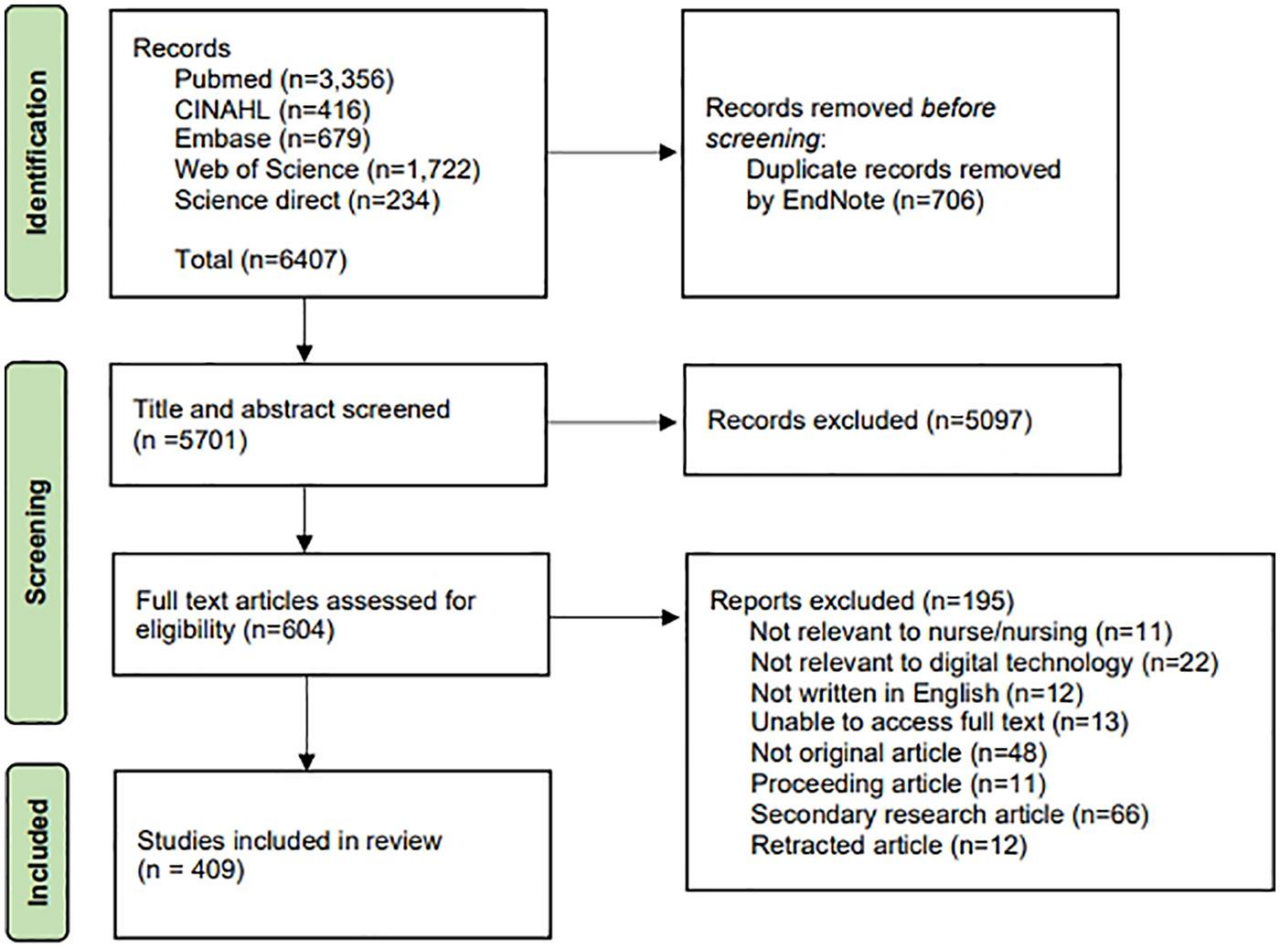
Article selection process.

The initial keyword search yielded 6,407 records. After removing 706 duplicates using EndNote, 5,701 articles were subjected to preliminary screening. Irrelevant items were excluded based on titles and abstracts. Subsequently, the full texts of 604 articles were assessed for eligibility, and 195 were excluded for reasons such as being non-original publications (editorials, commentaries, and letters to the editor), secondary research articles (systematic reviews, meta-analyses, and bibliometric studies), and ineligible publications (proceedings and retracted articles). Studies without accessible full texts were also excluded as their verification could not be performed. Finally, 409 articles were included in the final analysis (Figure 1).

### 2.3 Data analysis

Microsoft Excel 2019 was used to analyze the number of publications by journal, year, and country. VOSviewer 1.6.20 (Leiden University, Leiden, Netherlands) was used to visualize network maps for authors and keywords and identify the most cited publications.

## 3 Results

### 3.1 Number of publications by year

**Figure 2:**
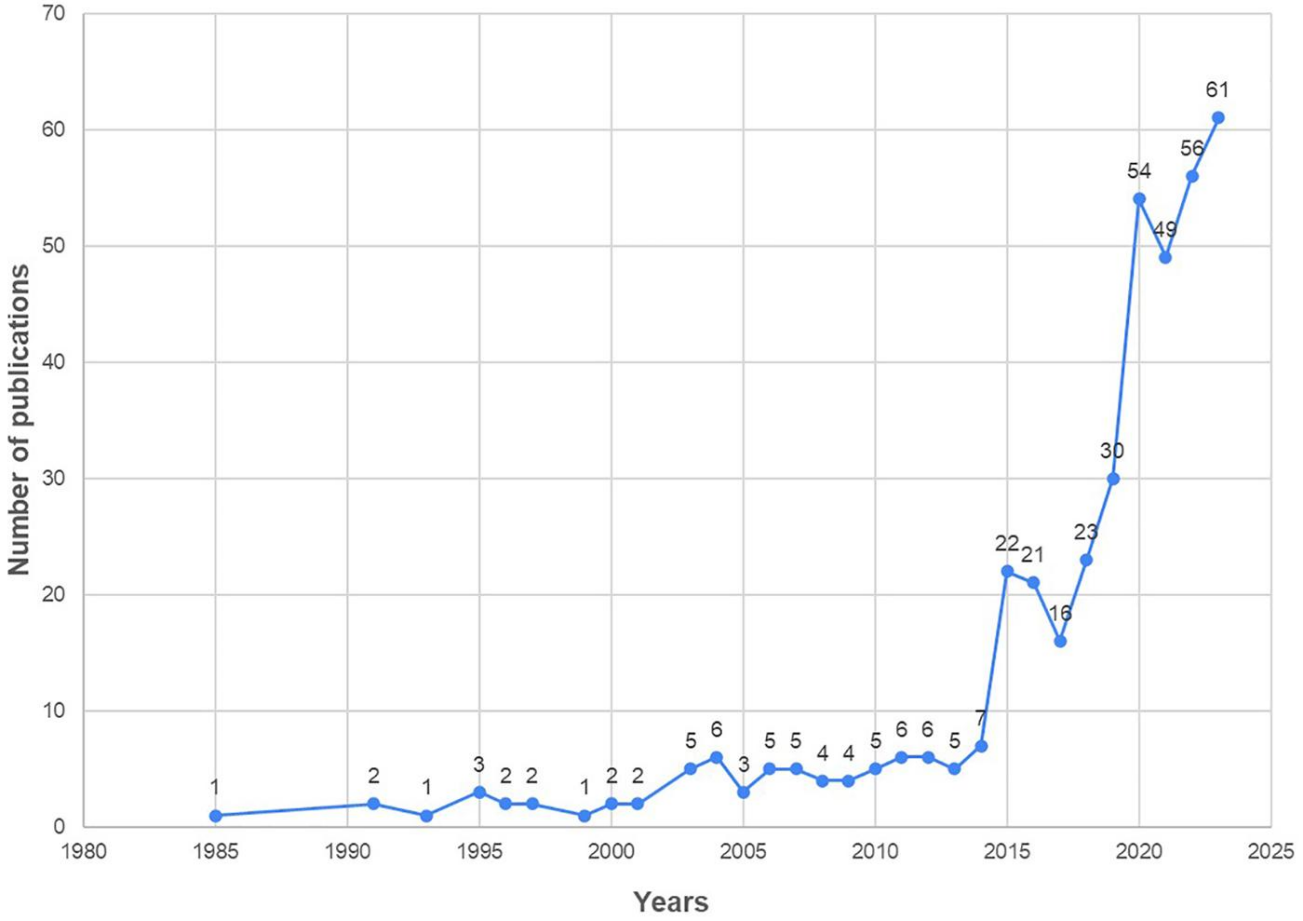
Number of publications by year.

The earliest recorded publication on digital technology in the field of nursing dates back to 1985. The highest number of publications was observed in 2023 (61 studies), followed by 2022 (56 studies) and 2020 (54 studies). Overall, the number of publications significantly increased from 2015, with a particularly notable surge from 30 studies in 2019 to 54 in 2020 (Figure 2).

### 3.2 Number of publications by country

**Figure 3:**
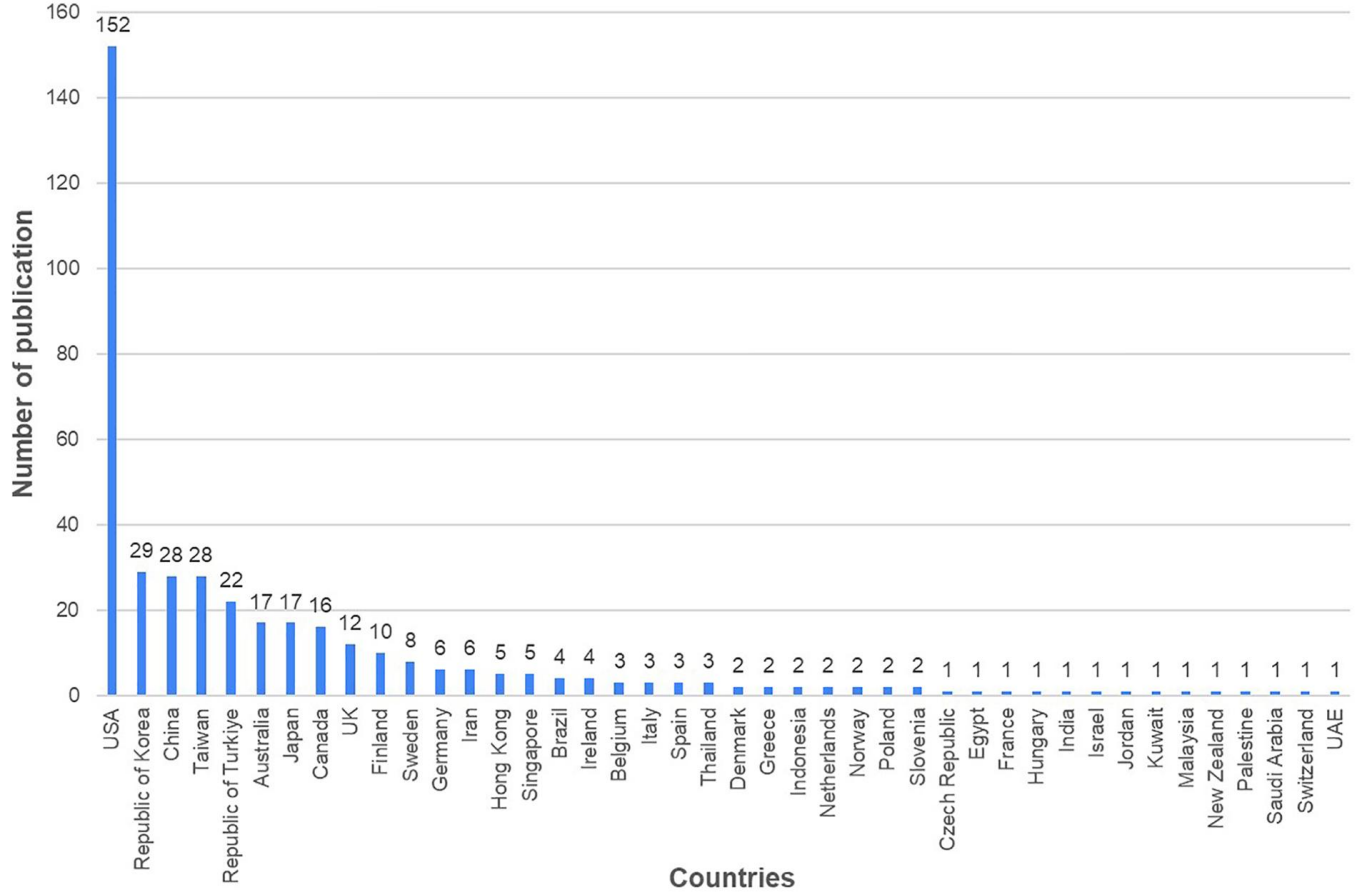
Number of publications by country. This figure shows the global distribution of research contributions in digital technology in nursing informatics. The country was assigned based on the corresponding author's affiliation.

The United States had the highest number of publications (152 times, 37.2%), followed by South Korea (29 times, 7.1%), China (28 times, 6.8%), and Taiwan (28 times, 6.8%). The top four countries collectively accounted for more than 50% of all the publications (Figure 3).

### 3.3 Publication journals

**Figure 4:**
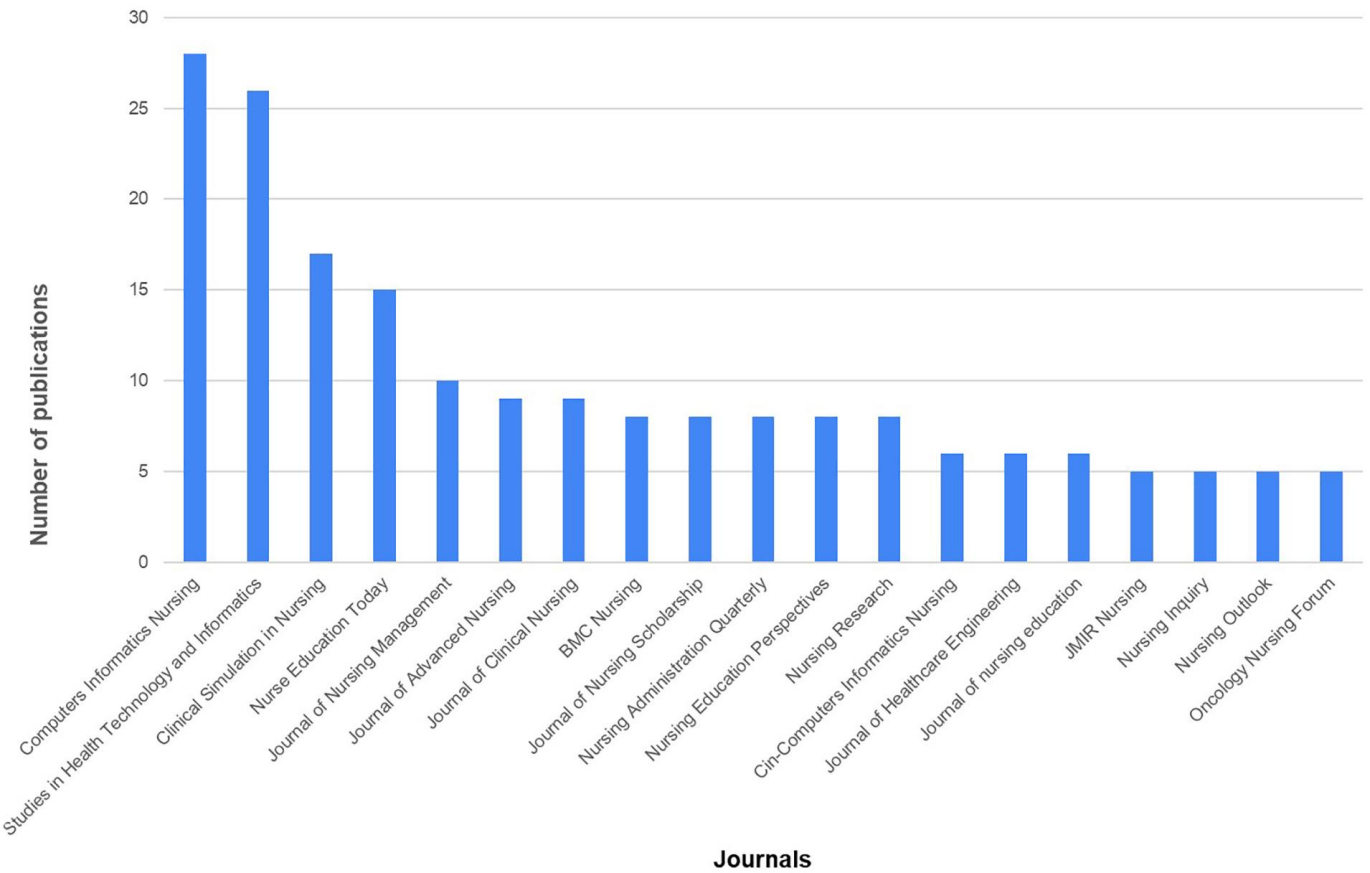
Number of publications by journal. This figure illustrates the journals with the highest frequency of publications in digital technology in nursing informatics. Only journals publishing five or more articles are included.

The journals with the highest number of publications were *Computers, Informatics, Nursing* (IF = 1.3), *Studies in Health Technology and Informatics* (SJR=0.29), *Clinical Simulation in Nursing* (IF = 3.4), *Nurse Education Today* (IF = 3.6), and *Journal of Nursing Management* (IF = 3.7). The publication counts of the top five journals accounted for 23.5% of the total publications (Figure 4).

### 3.4 Authors and co-citations

**Figure 5:**
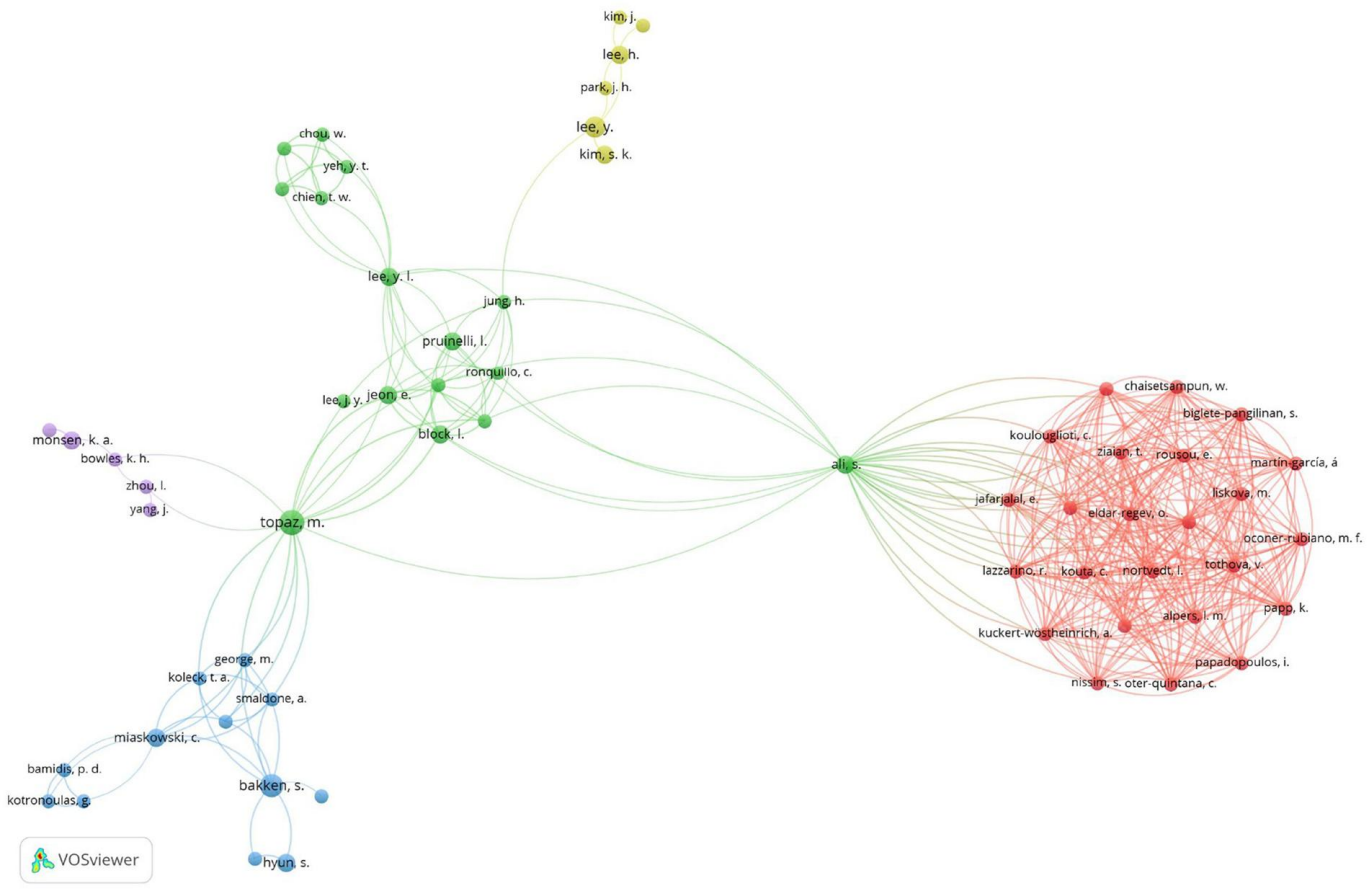
Network map of cooperation among authors.

A total of 1,612 authors were identified, of which only 179 published two or more articles. The researchers who published the most articles were Topaz M. (six articles), Hodgson E (six articles), Locsin R (six articles), Bakken S (five articles), Farra S (five articles), and Westra B (five articles). An analysis of co-citations among the 179 authors who published two or more articles revealed that 63 authors formed collaborative links. The network was primarily centered around Topaz M and Ali S (Figure 5).

### 3.5 Co-occurrence of keywords

A total of 1,186 keywords were identified, and a co-occurrence analysis was conducted on 47 keywords mentioned five or more times. The most frequently mentioned key terms were the following: virtual reality (85 mentions, connection strength 121); nursing (64 mentions, connection strength 109); AI (55 mentions, connection strength 57); machine learning (31 mentions, connection strength 34); nursing education (27 mentions, connection strength 48); simulation (24 mentions, connection strength 48); data mining (24 mentions, connection strength 19); nursing informatics (21 mentions, connection strength 29).

**Figure 6:**
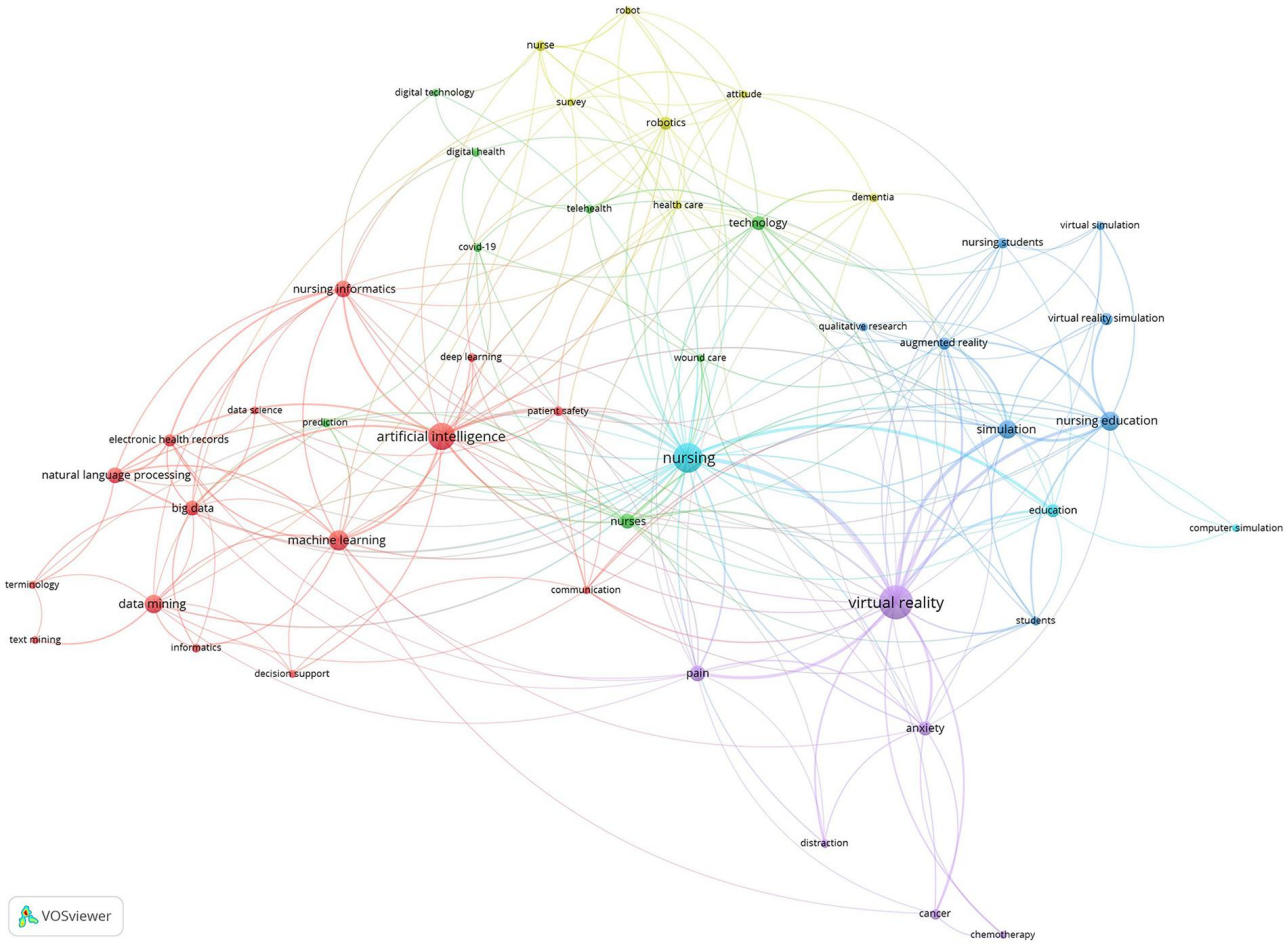
Co-occurrence of keywords.

The keywords formed six clusters (Figure 6):

- Cluster 1: Data utilization technologies.

This cluster comprised 15 keywords: AI, big data, data mining, data science, decision support, deep learning, electronic health record, informatics, machine learning, natural language process, nursing informatics, patient safety, terminology, text mining. These keywords collectively represent data-driven innovations in nursing, emphasizing the integration of advanced analytics and informatics for decision support and enhancing patient safety.

- Cluster 2: Digital and telehealth technologies.

This cluster comprised eight keywords: *COVID-19, digital health, digital technology, nurses, prediction, technology, telehealth, and wound care*. These technologies have been predominantly utilized in the context of social isolation and the COVID-19 pandemic, enabling the remote delivery of healthcare services. In particular, the inclusion of “wound care” reflects the growing field of telewound care.

- Cluster 3: Immersive learning technologies in nursing education.

This cluster comprised eight keywords: augmented reality, nursing education, nursing students, qualitative research, simulation, students, VR simulator, and virtual simulation. These keywords collectively highlight the growing application of immersive technologies, such as virtual and augmented reality, in nursing education.

- Cluster 4: Expansion of robotics and attitudes in nursing.

This cluster comprised seven keywords: *attitude, dementia, health care, nurse, robot, robotics, and survey*. Studies grouped within this cluster reflect the expanding use of robotic technologies in nursing practice and the growing attention to the acceptance and attitudes of nursing robotics.

- Cluster 5: VR-based nursing interventions for pain and anxiety management.

This cluster comprised six keywords*: anxiety, cancer, chemotherapy, distraction, pain, and VR*. This reflects nursing intervention studies that applied VR to patients with cancer, particularly those undergoing chemotherapy, to provide distraction from the experiences of pain and anxiety.

- Cluster 6: Computer simulation in nursing education.

This cluster comprised three keywords: *computer simulation, education, and nursing*. This study focuses on the use of computer-based simulation technologies in nursing education.

**Figure 7:**
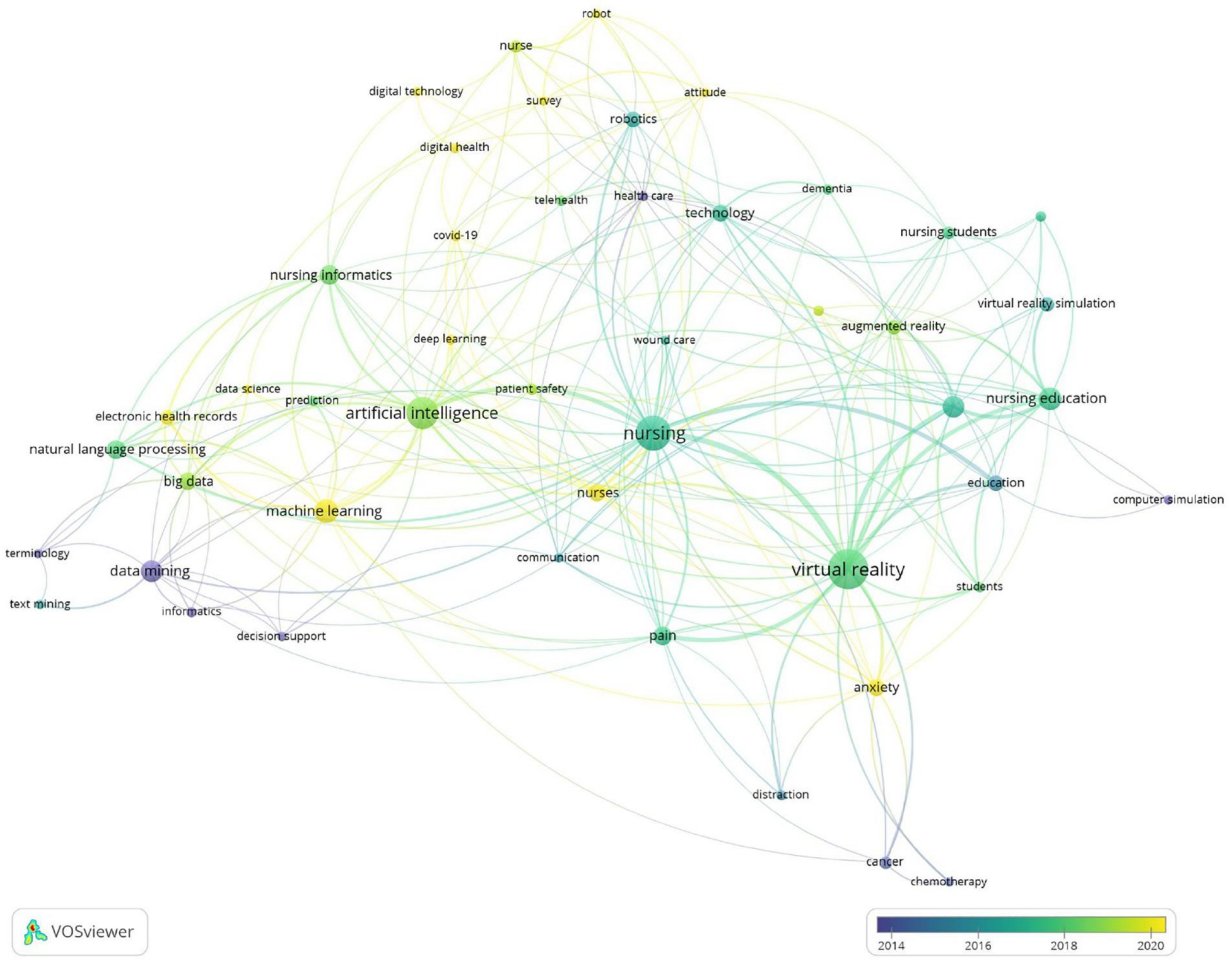
Visualization of keywords by the time when the keyword appeared.

From the analysis by year, the most recently used key terms were “machine learning,” “COVID-19,” “deep learning,” “anxiety,” and “electronic health records” (Figure 7).

### 3.6 Top 10 cited articles

Among the 409 publications, the most cited article was “A pilot and feasibility study of virtual reality as a distraction for children with cancer” by Gershon et al. (21), with 277 citations. Most of the top 10 most-cited articles indicate that, overall, virtual reality and digital health technologies have been actively studied in two main areas: nursing intervention for pediatric patients in clinical nursing and nursing education and training for nursing students (Table 1).

**Table 1:** Top 10 cited articles.

No.	Authors	Year	Title	Source	# of citation	IF/SJR of the year	FWCI
1	Gershon, J.; Zimand, E.; Pickering, M.; Rothbaum, B. O.; Hodges, L. (21)	2004	A pilot and feasibility study of virtual reality as a distraction for children with cancer	Journal of the American Academy of Child & Adolescent Psychiatry	277	3.529	2.33
2	Duan, L.; Street, W. N.; Xu, E. (36)	2011	Healthcare information systems: data mining methods in the creation of a clinical recommender system	Enterprise Information Systems	252	3.684	9.14
3	Libin, A.; Cohen-Mansfield, J. (37)	2004	Therapeutic robocat for nursing home residents with dementia: preliminary inquiry	American Journal of Alzheimer's Disease & Other Dementias	207	0.328	1.68
4	Schneider, S. M.; Workman, M. L. (29)	2000	Virtual reality as a distraction intervention for older children receiving chemotherapy	Pediatric Nursing	165	0.196	3.77
5	Nilsson, S; Finnström, B; Kokinsky, E; Enskär, K. (30)	2009	The use of Virtual Reality for needle-related procedural pain and distress in children and adolescents in a paediatric oncology unit	European Journal of Oncology Nursing	156	1.126	1.96
6	Brennan, P. F.; Bakken, S. (38)	2015	Nursing Needs Big Data and Big Data Needs Nursing	Journal of Nursing Scholarship	151	2.128	14.82
7	Butt, A. L.; Kardong-Edgren, S.; Ellertson, A. (33)	2018	Using Game-Based Virtual Reality with Haptics for Skill Acquisition	Clinical Simulation in Nursing	148	2.286	10.39
8	Schneider, S. M.; Hood, L. E. (39)	2007	Virtual reality: a distraction intervention for chemotherapy	Oncology Nursing Forum	139	1.438	1.33
9	Dubovi, I.; Levy, S. T.; Dagan, E. (40)	2017	Now I know how! The learning process of medication administration among nursing students with non-immersive desktop virtual reality simulation	Computers & Education	132	4.538	4.62
10	Hua, Y.; Qiu, R.; Yao, W. Y.; Zhang, Q.; Chen, X. L. (41)	2015	The Effect of Virtual Reality Distraction on Pain Relief During Dressing Changes in Children with Chronic Wounds on Lower Limbs	Pain Management Nursing	128	1.745	1.87

## 4 Discussion

This study used a bibliometric analysis based on the characteristics of the literature, utilizing a statistical method to analyze the distribution structure and quantitative relationship to inform future development (18, 22). The findings revealed the research trends and characteristics of applying digital technologies in nursing informatics: a significant increase in the number of publications from 2015; the highest number of publications in the United States and in the Journal of *Computer, Informatics, Nursing*; the research collaboration network centered on the two authors; six clusters among the co-occurrence keywords; and the dominance of virtual reality intervention as a research topic among the top 10 cited studies.

Over the literature review period, this study's findings showed that articles related to digital technology in nursing informatics have been published since 1985, and that the number of articles significantly increased from 2015 to 2020. In 1985, AI gained attention in healthcare, with a growing interest in its potential to enhance clinical decision-making (23). In 2015, nursing informatics research in acute care was conducted, with electronic health records and clinical systems being the most commonly used (24). In 2020, the COVID-19 pandemic was likely to contribute to this growing research trend. During the pandemic, when allocating limited medical resources, predictive models that assess the likelihood of adverse outcomes in infected patients based on pre-diagnosis data could facilitate the efficient triage of the patients (25) while minimizing physical contact with the patients. These contextual influences are presumed to have contributed to the significant increase in research on digital technology in nursing informatics.

The United States ranks first in terms of the number of publications. This indicates that the United States dominates studies on digital technology in nursing informatics, which may be due to the presence of a diverse patient population and extensive financial support for researchers (26). *Computer Informatics Nursing, Studies in Health Technology and Informatics, Clinical Simulation in Nursing, Nurse Education Today, and Journal of Nursing Management* published the highest number of articles. These journals likely focused on the intersection of nursing practice and healthcare technology, and published research on the application of informatics, education, and management strategies to enhance patient care and nursing outcomes. *Studies in Health Technology and Informatics* are not in nursing journals, but cover health informatics, such as healthcare technology, data management, and digital health innovations. Therefore, nursing researchers should focus on these journals for the lastest multidisciplinary research.

The most prolific author of reviewed articles was Topaz M with six publications. A collaborative research network was established, primarily centered on Topaz M and Ali S. Topaz et al. completed their research with multidisciplinary researchers from medical and nursing schools (27), nursing, data science, and biometric informatics (28). In particular, the network analysis of the co-cited authors showed a well-organized research collaboration structure with strong interconnections centered around Ali S, who conducted research with researchers from multiple institutions, indicating an effective knowledge-sharing cluster consistent with previous findings on co-authorship networks (26). These studies highlighted the importance of multidisciplinary cooperation in this field. Therefore, researchers in nursing informatics should be encouraged to participate in multidisciplinary research collaborations related to AI and digital technologies.

Hot topics, based on the distribution of high-frequency keyword analysis of co-occurring keywords, highlighted the potential applications of virtual reality and AI in nursing informatics. Cluster analysis of these keywords revealed that digital technology is being utilized in education and interventions, which indicates that researchers have focused on the application of virtual reality in nursing education and simulation-based training interventions. As previously described, the ten most frequently cited studies identified by our analysis also investigated nursing intervention programs utilizing virtual reality as an effective distraction method during invasive treatment for patients (29, 30) and as a tool to enhance nursing professionals’ understanding of clinical situations (31). Interest in virtual reality arises from its immersive and participatory nature. Virtual reality is a computer-stimulated practice that allows participants to hear and watch stimuli corresponding to a visual image (29). Researchers have also utilized AI technologies such as machine learning and data mining. There is no doubt that machine learning methods to improve clinical decision-making algorithms represents one of the most advanced and applicable domains of AI in nursing informatics (26).

When analyzing the co-citations of references, this study's findings showed that the study entitled “A pilot and feasibility study of virtual reality as a distraction for children with cancer” was the most cited (277 times) and was published in *Journal of the American Academy of Child & Adolescent Psychiatry* by Gershon et al. (21). This study demonstrated that virtual reality is an effective distraction intervention for pediatric cancer patients to alleviate distress and anxiety during invasive medical procedures. This reflects the potential of virtual reality technology as an informatics-driven intervention in pediatric oncology nursing and emphasizes its role in enhancing patient-centered care by reducing procedural anxiety. The fact that this pilot and feasibility study examined virtual reality in terms of safety, acceptability, and feasibility at a time when virtual reality technology was still under development in the medical and nursing fields may have contributed to its high citation count. Even today, it is cited in review articles on the impact of virtual reality as a psychological variable in pediatric cancer patients (32), making it a pioneering work in this field.

Consistent with our findings, highlighting the role of nursing professionals as practitioners and active participants is a common feature of AI nursing research (8, 26). This is because nursing professionals have the advantage of possessing a profound knowledge of patients’ needs. Hence, nursing professionals should be technically skilled in actively participating in digital technology in nursing informatics research and practice. Although nursing students have already received education related to e-learning and information communication technology (33), there is a pressing need for educational programs of digital technology competency targeting nurses currently working in various clinical settings.

Overall, the nursing informatics literature discussing and examining various technologies used by nurses in practice, leadership, research, and education has been analyzed in this study, but there is an underexplored area of developing nursing practice standards for digital technology applications. The exponential proliferation of nascent digital technologies has generated a state of ambiguity regarding how best to align contemporary nursing roles and knowledge with healthcare in the imminent future (13). For instance, how nurses should consider patients’ use of virtual spaces in their assessment of patient health, and subsequent nursing interventions must be discussed (34). To achieve this, deeper exploration and reconceptualization of nurses’ responsibilities and expertise in future healthcare interventions are required (13). With no established consensus, nurses’ professional identities and ethical considerations surrounding the application of digital technology in nursing informatics also remain an ongoing topic of discussion (35). Further studies are required to explore these aspects.

Although the literature search was extensive within this review, only the studies written in English were included in the analysis. Studies from technologically advanced countries that were not written in English were excluded from the review. Future research could utilize digitally innovative translators to examine literature written in languages other than English, thereby enabling accurate cross-national comparisons and providing a detailed understanding of research networks and trends related to digital capabilities and the quality of care in nursing informatics.

## 5 Conclusions

This bibliometric analysis highlights the growing significance of digital technology from the perspective of nursing informatics. The surge in research activities since 2015, driven by the advent of AI, virtual reality, and big data analytics, has underscored the increasing recognition of digital innovations as pivotal to advancing nursing practices and education. Despite notable progress, unexplored areas such as non-standardized nursing implementation using digital technologies and ethical considerations remain. Addressing these gaps requires concerted efforts to integrate interdisciplinary collaboration, enhance technical competencies among nursing professionals, and establish robust standards for the ethical application of digital technologies. Future research should focus on exploring underrepresented areas, fostering global collaboration, and developing evidence-based practices to optimize the benefits from applications of digital technology in nursing informatics.

## 6 Implications for nursing informatics

From the perspective of nursing informatics, multidisciplinary research collaboration networks should be encouraged at the level of educational institutions and academic societies. As the effectiveness of virtual reality and AI is increasingly demonstrated in nursing education and practice, there is an urgent need to enhance nurses’ competencies related to digital technologies by investing in targeted training programs using virtual reality or AI. This will reinforce the role of nurses as coordinators between the patient experience and innovative technologies. In addition, standards of nursing informatics for care utilizing digital technologies must be provided to minimize clinical challenges or role conflict. Nursing informatics researchers and specialists should focus on these areas to navigate the direction of digital technologies in nursing.

## Data Availability

The original contributions presented in the study are included in the article/Supplementary Material, further inquiries can be directed to the corresponding author.
