# Supplementary material for: Trends in application of digital technology in nursing informatics: an integrative bibliometric analysis
Source: Front Digit Health. 2026 Feb 23;8:1670402. doi: 10.3389/fdgth.2026.1670402 (PMC12968252; doi:10.3389/fdgth.2026.1670402)
Supplement: Supplementary file 1 [file Datasheet1.docx]

***Supplementary Material***

# Supplementary Data (Appendix A. The detailed search strategy)

TS=("Artificial Intelligence" OR "AI" OR "Computational Intelligence" OR "Machine Intelligence" OR "Machine learning" OR "Knowledge Representations" OR "Deep Learning" OR "Natural Language Processing" OR "Neural Network" OR "Autonomous Robot" OR "Automatic Programming" OR "Intelligent Tutoring" OR "Intelligent Agents" OR OR "Voice Recognition" OR "Text Mining" OR "ChatGPT" OR "Chatbot" OR "Digital" OR "Big data" OR "Drone" OR "Augmented reality" OR "Virtual reality" OR "Metaverse" OR "Internet of things") AND (TS=("Nursing" OR "Nurse") OR "Nurs* Care") OR "Nursing Informatics").
